# Patients’ choice of healthcare provider for primary hip and knee arthroplasty: a national register-based observational cohort study of 62,676 patients from Norway, 2014–2018

**DOI:** 10.2340/17453674.2026.46430

**Published:** 2026-08-31

**Authors:** Beate HAUGLANN, Cato KJÆRVIK, Eva STENSLAND, Yohannes TESFAY, Anne Marie FENSTAD, Ove FURNES, Tor INGEBRIGTSEN

**Affiliations:** 1 Centre for Clinical Documentation and Evaluation (SKDE), Northern Norway Regional Health Authority, Tromsø; 2 Department of Surgery, Nordland Hospital Trust, Vesteraalen Hospital, Stokmarknes; 3 Department of Clinical Medicine, Faculty of Health Science, UiT The Arctic University of Norway, Tromsø; 4 Department of Community Medicine, Faculty of Health Science, UiT The Arctic University of Norway, Tromsø; 5 The Norwegian Arthroplasty Register, Department of Orthopaedic Surgery, Haukeland University Hospital, Bergen; 6 Department of Clinical Medicine, Faculty of Medicine, University of Bergen, Bergen; 7 Department of Neurosurgery, Ophthalmology and Otorhinolaryngology, University Hospital of North Norway, Tromsø, Norway

## Abstract

**Background and purpose:**

We aimed to quantify how provider-related factors are associated with patients’ revealed, GP-mediated choice of arthroplasty provider in Norway, expressed as willingness to travel (WTT) in minutes, and to explore how WTT for travel time was modified by patient characteristics.

**Methods:**

This is a national observational cohort study including all publicly funded, elective primary hip and knee arthroplasties in Norway, 2014–2018 (n = 62,676), with linkage to national registries. A mixed logit model was used to analyze choice among providers with different attributes, in both preference and WTT space. Associations between individual-level travel-time preferences and patient characteristics were analyzed with multilevel modelling.

**Results:**

Patients accepted substantial additional travel time for several provider attributes. Mean WTT was 156 min (95% confidence interval [CI] 130–182; median 8) for a dedicated elective surgical unit, 70 min (CI 67–72) for a provider within the patient’s regional health authority, 16 min (CI 15–17) for above-median surgical volume, 4 min (CI 3–5) for an adult child living near the provider, and 3 min (CI 3–3) for below-median waiting time. WTT for travel time was reduced in patients with high comorbidity (−27%), low education (−33%), and low income (−30%), with graded gradients across categories, and decreased with increasing age.

**Conclusion:**

Provider attributes were associated with patients’ revealed, GP-mediated choice of arthroplasty provider, expressed as clinically interpretable WTT. WTT was lower among older, sicker, and socioeconomically disadvantaged patients, indicating that realized choice is patterned by both modifiable structural features of the healthcare system and patient-level socioeconomic gradients.

Several publicly funded European healthcare systems allow patients the choice of provider for elective healthcare [1,2]. This policy aims to enhance efficiency and quality of care through competition [3,4]. Norway has offered patient choice since 2001 [5].

A Norwegian qualitative study showed that general practitioner (GP) referrals for arthroplasty reflect a complex interplay of patient expectations, hospital reputation, and logistical constraints [6]. Elective hospital care requires a GP referral, so autonomous patient choice varies. Reviews show patients consider structural, process, and outcome factors when choosing providers [1]. Proximity to the provider is the primary determinant [7]. Metropolitan patients often have several providers nearby, unlike rural patients. It has been shown that high patient mobility was associated with provider characteristics such as shorter waiting times, indicators of better quality, and more advanced treatment options, but low with high age and low socioeconomic status [3,8].

The primary aim of our study was to quantify how provider-related factors are associated with revealed patients’ preferences of hospital for primary hip or knee arthroplasty in Norway, expressed as marginal utility and willingness to travel (WTT) in minutes for a given provider characteristic. As a secondary aim, we examined how WTT for travel time was modified by patient characteristics.

## Methods

### Study design and setting

This is a national observational cohort study using prospectively collected register data for Norway (5.3 million inhabitants in 2018), reported according to the REporting of studies Conducted using Observational Routinely collected health Data (RECORD) guidelines.

In Norway, 4 regional health authorities provide specialized care via local hospitals or contracted private providers in a universal, tax-based single-payer system (travel costs are mostly covered).

Publicly funded hip and knee arthroplasty was provided by 45 public and 6 private non-profit hospitals during the study period. Private non-profit capacity was concentrated in the west and south-east, mostly in the capital area [5,9]. The Norwegian Health Atlas showed geographic variation in treatment rates for hip and knee arthroplasty and in whether patients received care from their local provider [10].

### Participants and data sources

We obtained individual-level data from 3 national registers: 1 medical quality registry (the Norwegian Arthroplasty Register [NAR]), 1 patient administrative registry (the Norwegian Patient Registry [NPR]), and the statistical institute (Statistics Norway).

All patients (≥ 18 years) residing in Norway who had publicly funded elective primary hip or knee arthroplasty between January 1, 2014, and December 31, 2018, were included.

The study population was primarily identified in data provided by the NAR. A small proportion not in the NAR were identified in NPR data using NCSP codes for primary hip (NFB20/30/40/99) and knee (NGB20/30/40/99) arthroplasty, with all ICD-10 codes included. Primary osteoarthritis (M16, M17) accounted for 92% of hips and 95% of knees; the remainder included all other diagnoses. Patients were tracked across all registries by their unique personal identity number. Data was linked using pseudonymized identifiers.

The NAR has collected data on hip and knee arthroplasties since 1987 and 1994, respectively, with persistently high (95%) capture rates for both [11,12]. Individual NPR data covered all public hospitals and contracted private hospital visits from January 2013 through December 2018. The index operation was defined as the first primary hip or knee arthroplasty within the inclusion period. Data included diagnoses (ICD-10 codes), procedures, hospital, and admission and discharge dates. Statistics Norway provided demographic and socioeconomic information, including residential details, education, and annual income, for patients and their adult children (≥ 18 years).

### Variables

We characterized arthroplasty providers by: (i) waiting time to surgery, (ii) annual surgical volume, and (iii) the presence of dedicated elective surgical units (orthopedic surgical capacity reserved for elective procedures and protected from emergency interruption). We also defined provider–patient contextual measures: (iv) patients’ travel time to each provider, (v) whether an adult child lived near the provider, and (vi) whether the provider was within the patient’s regional health authority.

Hospitals report estimated waiting times to the Norwegian Directorate of Health. We dichotomized waiting times at the median into short (< 10.2 weeks) and long (> 10.2 weeks). Providers’ average annual surgical volume of hip or knee arthroplasties per year in the study period was retrieved from the NAR annual reports [11] and dichotomized at the median into high (> 109) and low (< 109). Most public hospitals share elective and emergency orthopedic resources, whereas private non-profit and a few public hospitals operate dedicated elective units. We therefore categorized each provider as having an elective surgical unit or not, based on information obtained by direct contact with the providers.

Travel times (in minutes) between the 428 municipal city halls and the 51 providers were defined as the fastest road and/or air transport time. The geographic location of the arthroplasty providers was classified as within or outside the patient’s regional health authority based on the patient’s municipal or city district residency in the year of surgery. “Adult child living nearby” was coded “yes” if the patient had ≥ 1 adult child residing within 45 road-minutes of the provider in the year of surgery, otherwise “no.”

Age at the time of surgery was a continuous variable. We measured comorbidity with a modified version of the Charlson Comorbidity Index (CCI) [13], based on diagnosis codes from hospitalizations within a 1-year look-back window. The comorbidity index was categorized into none (CCI = 0), medium (CCI = 1–2), and high (CCI ≥ 3). Educational levels according to the International Standard Classification of Education were recoded into low (primary school), medium (upper secondary school), and high (undergraduate and postgraduate education) [14]. Total after-tax personal income in the year before the year of surgery was index-adjusted to 2015 to account for inflation and divided into quartiles: low, medium-low, medium-high, and high.

### Outcomes

The outcome was the patient’s observed choice of arthroplasty provider for the index operation among the 51 available providers. From the mixed logit model of this choice, we derived 2 measures for each provider attribute: the marginal utility, expressing the strength of the attribute’s association with provider choice, and the willingness to travel (WTT), defined as the additional travel time in minutes associated with the attribute, calculated as the ratio of the attribute’s marginal utility to the marginal disutility of 1 additional minute of travel. WTT was not directly elicited from patients; it is an estimate inferred from observed, GP-mediated provider choices and should be interpreted as revealed rather than stated willingness. The secondary outcome was the individual-level marginal utility of travel time derived from the mixed logit model, used in the analyses of modification by patient characteristics.

### Statistics

Categorical variables are presented as absolute numbers and percentages. Continuous variables were handled as follows: age at surgery was retained as a continuous covariate and modelled using natural splines to allow for non-linear effects; in descriptive statistics, age is reported as median and interquartile range. Travel time (in minutes) from the patient’s municipality to each provider was treated as a continuous attribute in the mixed logit model.

Missing data was limited. Small amounts of missingness in education (1.4%) and income (0.2%) are reported descriptively. Missing values were coded as separate levels for each variable, with estimates for these levels omitted from the tables. The results did not materially change when analyses excluded observations with missing data.

The choice of provider among a set of alternatives was investigated using discrete choice models in both the preference (marginal utility) and WTT space. The preference space approach estimates how much patients value each hospital attribute when choosing provider. The WTT space converts the preferences into minutes of travel time associated with a provider attribute. This reflects the change in travel time required to keep the overall utility constant when the provider attribute changes. In discrete choice, patients are assumed to maximize utility based on latent preferences [15]; we inferred revealed preferences from observed provider choices. The mixed logit model was selected due to its capability to account for heterogeneity in preferences through random coefficients [15]. Travel time was modelled with a negative log-normal distribution (constraining the effect to be negative for all patients) and dedicated elective surgical unit with a log-normal distribution (constraining it to be positive), reflecting expected desirability. Adult child nearby, waiting time, surgical volume, and within-region treatment were modelled as fixed parameters with the same average effect across patients. We present 95% confidence intervals (CIs) and standard deviations (SD) where appropriate.

Subsequent analyses of associations between revealed preferences for travel time to the provider and patient characteristics involved the centered individual-level marginal utilities for travel time (i.e., each patient’s travel-time parameter) derived from the mixed logit model. These were further analyzed with multilevel modelling [16] across nested municipality and health enterprise levels to account for the hierarchical structure. Income, education, sex, comorbidity, and type of arthroplasty were included as fixed effects, while age was modelled using natural splines. Individual coefficients were mean-centered. Descriptive analyses were done in SAS v.8.3 (SAS Institute, Cary, NC, USA); mixed logit (Apollo 0.3.7) and multilevel (lme4 1.1.34) models were fitted in R 4.6.0 [17-20] (R Foundation for Statistical Computing, Vienna, Austria).

### Ethics, data sharing plan, funding, and disclosures

The Regional Committee for Medical and Health Research Ethics (REK) North determined the study to be outside the scope of the Health Research Act and therefore it did not require ethical approval (ref. 2018/1955). REK and Statistics Norway granted exemptions from the duty of confidentiality for the use of data obtained from the NPR and Statistics Norway, respectively. Data recorded in the NAR is collected with informed consent. The Northern Norway Regional Health Authority’s data protection officer confirmed compliance with Norwegian and EU regulations, and a data protection impact assessment (DPIA) was completed. Data was obtained following formal applications to the NAR, the Norwegian Health Directorate, and Statistics Norway. Public access to this data is restricted.

This study was performed as part of the authors’ employment at their respective institutions, and no external project funding was received.

The authors declare no conflicts of interest. Complete disclosure of interest forms according to ICMJE are available on the article page, doi: 10.2340/17453674.2026.46430

## Results

**Figure 1:**
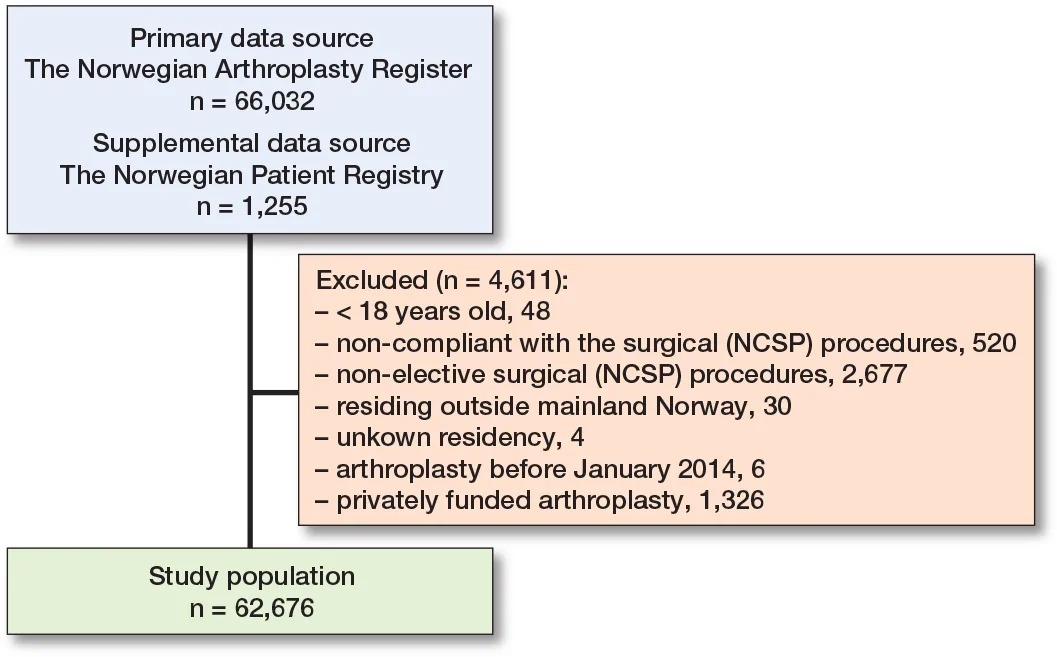
Flowchart.

62,676 adult patients (≥ 18 years) had publicly funded elective primary hip or knee arthroplasty in Norway between January 1, 2014, and December 31, 2018 (Table 1). The NAR had information on 61,704 patients, and the remaining 972 patients were identified from the NPR. The flowchart shows the number of patients identified by the NAR and the NPR, and the exclusions (Figure 1).

**Table 1:** Characteristics of patients with publicly funded elective primary hip or knee arthroplasty in Norway, 2014–2018. Values are count (%)

Factor	Total population	Choice of provider	
		Within health region	Outside health region
Total sample	62,676	58,990 (94)	3,686 (5.9)
Sex			
Male	23,884 (38)	22,213 (93)	1,671 (7.0)
Female	38,792 (62)	36,777 (95)	2,015 (5.2)
Age, median (IQR)	69 (62–76)	69 (62–76)	67 (60–73)
Arthroplasty			
Knee	26,595 (42)	25,126 (94)	1,469 (5.5)
Hip	36,081 (58)	33,864 (94)	2,217 (6.1)
Comorbidity			
High (CCI ≥3)	1,344 (2.1)	1,297 (97)	47 (3.5)
Medium (CCI 1–2)	9,056 (14)	8,632 (95)	424 (4.7)
None	52,276 (84)	49,061 (94)	3,215 (6.2)
Education **^a^**			
Low	15,199 (24)	14,502 (95)	697 (4.6)
Medium	31,436 (50)	29,561 (94)	1,875 (6.0)
High	15,168 (24)	14,106 (93)	1,062 (7.0)
Missing data	873 (1.4)	821 (94)	52 (6.0)
Personal net income			
Low (Q1)	15,906 (25)	15,248 (96)	658 (4.1)
Medium low (Q2)	15,896 (25)	15,066 (95)	830 (5.2)
Medium high (Q3)	15,745 (25)	14,730 (94)	1,015 (6.4)
High (Q4)	15,016 (24)	13,845 (92)	1,171 (7.8)
Missing data	113 (0.2)	101 (89)	12 (11)
Children			
No	6,284 (10)	5,944 (95)	340 (5.4)
Yes	56,392 (90)	53,046 (94)	3,346 (5.9)
Referral area of health enterprise			
Finnmark	798 (1.3)	585 (73)	213 (27)
UNN	2,268 (3.6)	1,729 (76)	539 (24)
Nordland	1,752 (2.8)	1,340 (77)	412 (24)
Helgeland	1,078 (1.7)	745 (69)	333 (31)
Nord-Trøndelag	2,123 (3.4)	1,789 (84)	334 (16)
St. Olavs Hospital	3,695 (5.9)	3,270 (89)	425 (12)
Møre og Romsdal	3,504 (5.6)	3,266 (93)	238 (6.8)
Førde	1,881 (3.0)	1,770 (94)	111 (5.9)
Bergen	4,606 (7.3)	4,542 (99)	64 (1.4)
Fonna	2,200 (3.5)	2,149 (98)	51 (2.3)
Stavanger	3,358 (5.4)	2,823 (84)	535 (16)
Østfold	3,744 (6.0)	3,721 (99)	23 (0.6)
Akershus	5,403 (8.6)	5,371 (99)	32 (0.6)
Innlandet	5,989 (9.6)	5,831 (97)	158 (2.6)
Vestre Viken	6,389 (10)	6,299 (99)	90 (1.4)
Vestfold	3,242 (5.2)	3,215 (99)	27 (0.8)
Telemark	2,322 (3.7)	2,306 (99)	16 (0.7)
Sørlandet	3,974 (6.3)	3,924 (99)	50 (1.3)
Oslo	4,350 (6.9)	4,315 (99)	35 (0.8)
Year of treatment			
2014	12,900 (21)	12,142 (94)	758 (5.9)
2015	12,500 (20)	11,823 (95)	677 (5.4)
2016	12,538 (20)	11,777 (94)	761 (6.1)
2017	12,256 (20)	11,562 (94)	694 (5.7)
2018	12,482 (20)	11,686 (94)	796 (6.4)

a Low = primary school; medium = upper secondary school; high = under- and postgraduate. UNN = University Hospital of North Norway.

### Patient characteristics

**Figure 2:**
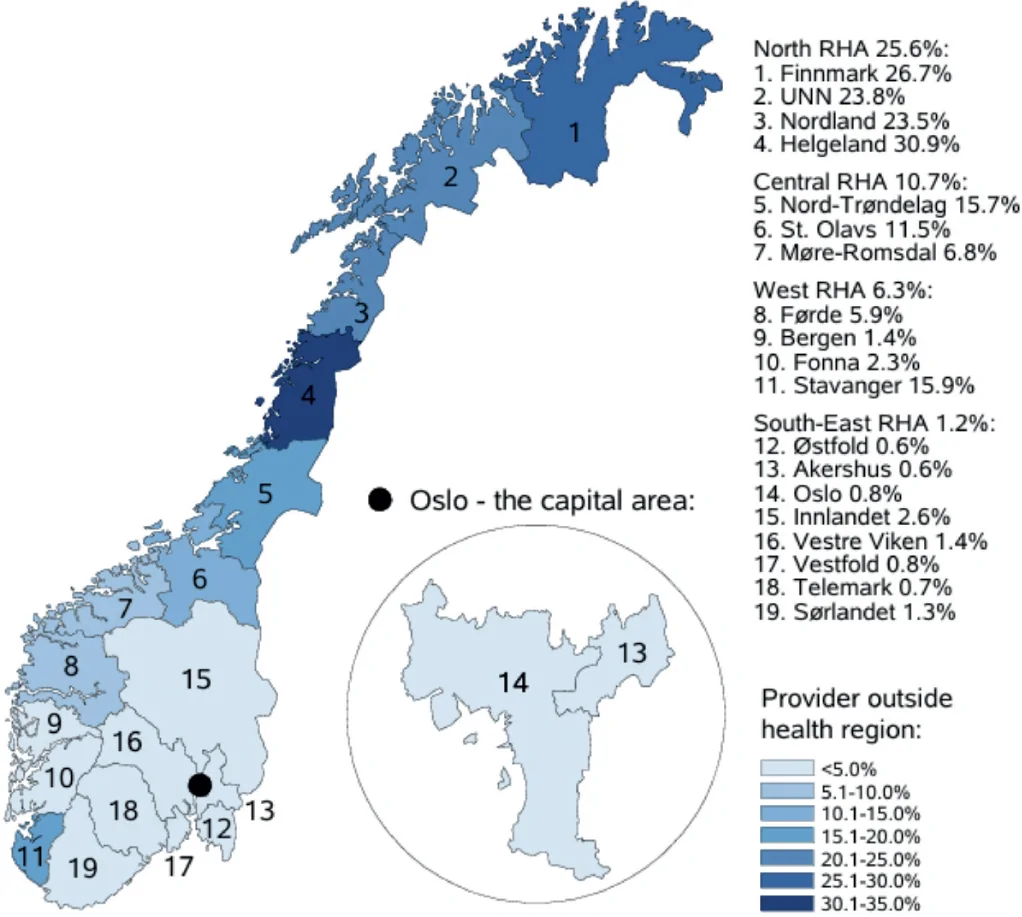
Proportion of patients receiving hip or knee arthroplasty from a provider outside the health region (RHA) of residence in the 19 health enterprise referral areas in Norway, 2014–2018. RHA = Regional Health Authority. UNN = University Hospital of North Norway.

Overall, 94% received treatment from an arthroplasty provider within, and 5.9% from a provider outside their health region (see Table 1). However, there was substantial geographic variation across the health regions and the referral areas of the health enterprises in the proportion of patients treated outside the region (Figure 2), ranging from 31% (Helgeland) to 0.6% (Akershus and Innlandet). Most patients were female (62%); median age was 69 years (IQR 62–76). Hip arthroplasty accounted for 58% and knee arthroplasty for 42%. Most patients (84%) had no recorded comorbidity measured by CCI. Regarding education, 50% had completed high school, and 24% had a college education.

### Provider-related factors in the choice of arthroplasty provider

A table of provider characteristics across the 51 arthroplasty hospitals is provided in Table S1 (see Supplementary data).

Additional travel time was associated with lower revealed preference for a provider, holding the other included provider attributes constant (Table 2). A substantial difference between patients in tolerance for travel time was revealed by the discrepancy in preference (marginal utility) per additional travel minute between the mean (−0.18, CI –0.19 to –0.17) and the median (−0.06). Values closer to zero indicate greater travel-time tolerance, whereas more negative values indicate lower tolerance.

**Table 2:** Results from the mixed logit model for provider-related factors reported in both the preference space and willingness to travel (WTT) space

Factors	Preference space marginal utility			WTT space WTT minutes	
	mean (CI)	median	SD (CI)	mean (CI)	median
Travel time (min) **^a^**	–0.18 (–0.19 to –0.17)	–0.06	0.52 (0.47–0.58)	–	
Elective surgical unit **^a^**	2.4 (2.2 to 2.6)	0.57	9.7 (7.6–12)	156 (130–182)	7.7
High surgical volume	1.0 (0.98 to 1.1)			16 (15–17)	
Short waiting time	0.20 (0.17 to 0.22)			2.9 (2.5–3.3)	
Within health region	2.2 (2.1 to 2.2)			70 (67–72)	
A child living nearby	0.59 (0.54 to 0.64)			3.9 (2.8–4.9)	

a Random coefficient.

CI = 95% confidence interval; SD = standard deviation.

The difference between the mean and median values in the travel-time results, together with the SD, suggests that the distribution is negatively skewed, with most probability mass near zero and a longer tail towards more negative values, consistent with substantial between-patient heterogeneity.

Between-patient variation was also present in the willingness to travel for elective surgical unit. The median WTT was 8 min, whereas the mean was 156 min (CI 130–182), implying that the typical patient was willing to accept only a little additional travel time, while a smaller subgroup had higher travel-time tolerance for elective surgical unit.

Arthroplasty at a hospital within the health region of residence was associated with higher willingness to travel compared with outside it (WTT 70, CI 67–72; marginal utility: 2.2, CI 2.1–2.2). High surgical volume (WTT 16, CI 15–17; marginal utility: 1.0, CI 0.98–1.1), short wait time (WTT 2.9, CI 2.5–3.3; marginal utility: 0.20, CI 0.17–0.22), and having a child living nearby the provider (WTT 3.9, CI 2.8–4.9; marginal utility: 0.59, CI 0.54–0.64) were also associated with higher willingness to travel and positive marginal utility, although these estimates were smaller.

### Patient characteristics associated with the utility of travel time

**Figure 3:**
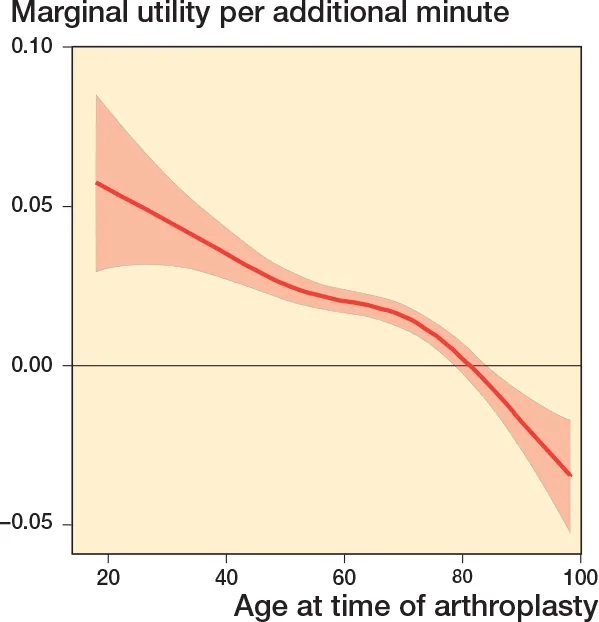
Association between age and individual-level travel-time utility of the multivariable model.

Between-patient heterogeneity in travel-time preference was associated with age, comorbidity, and socioeconomic status, but not with sex or joint type after adjustment (Table 3). Marginal utility decreased with age, becoming more negative around 67 years (Figure 3), indicating lower willingness to accept additional travel time with increasing age.

**Table 3:** Individual factors affecting patient preference for travel time (marginal utility of travel time) to the provider for hip and knee arthroplasty per additional minute of travel

Factor	Univariate model (CI)	Multivariate model (CI)	Change (%) in willingness to travel
Sex (ref.: female)			
Male	0.010 (0.006 to 0.013)	–0.001 (–0.003 to 0.004)	
Arthroplasty (ref.: hip)			
Knee	0.003 (0.000 to 0.007)	0.001 (–0.001 to 0.004)	
Comorbidity (ref.: CCI 0)			
High (CCI ≥ 3)	–0.034 (–0.045 to –0.023)	–0.022 (–0.032 to –0.015)	–27
Medium (CCI 1–2)	–0.014 (–0.019 to –0.010)	–0.007 (–0.011 to –0.002)	–10
Education (ref.: high)			
Low	–0.032 (–0.036 to –0.029)	–0.030 (–0.035 to –0.025)	–33
Medium	–0.015 (–0.019 to –0.011)	–0.018 (–0.026 to –0.014)	–23
Income (ref.: high Q4)			
Low (Q1)	–0.033 (–0.038 to –0.029)	–0.022 (–0.027 to –0.016)	–30
Medium low (Q2)	–0.025 (–0.029 to –0.021)	–0.018 (–0.022 to –0.013)	–23
Medium high (Q3)	–0.012 (–0.016 to –0.007)	–0.009 (–0.013 to –0.005)	–13

CI = 95% confidence interval.

A natural spline for age and municipal residency nested within the referral area of the health enterprise was applied in the multivariable model.

From the multivariable model, change (%) in willingness to travel compared with the reference groups of comorbidity, education, and income was estimated based on the median travel-time marginal utility from the mixed logit model as the reference value.

Comorbidity showed a graded association with travel-time preference, with a progressive reduction for the medium (–0.007, CI –0.011 to –0.002) and high (–0.022, CI –0.032 to –0.015) comorbidity groups compared with the no comorbidity group.

Using the median travel-time marginal utility from the mixed logit model as the reference value (–0.06), this corresponds to approximately 27% reduction in the high comorbidity group and 10% reduction in the medium comorbidity group in willingness to travel for improvements in provider-related factors compared with the no comorbidity group. Increasing comorbidity was thus associated with progressively lower willingness to travel. Similarly, socioeconomic factors also showed graded associations with travel time preference. Compared with patients in the high education group, marginal utility was lower among patients with medium (–0.018, CI –0.026 to –0.014) and low (–0.030, CI –0.035 to –0.025) education. The same trend was observed across the income categories. The travel time preference of patients in medium-high (–0.009, CI –0.013 to –0.005), medium-low (–0.018, CI –0.022 to –0.013), and low (–0.022, CI –0.027 to –0.016) income groups were progressively lower compared with patients in the high-income group. Taking the median travel-time marginal utility from the mixed logit model as the reference value, these estimates correspond to approximately 23% lower willingness to travel in the medium education group and 33% lower willingness to travel in the low education group for the same improvement in provider-related factors. The corresponding reductions across income categories were approximately 13%, 23%, and 27% for the medium-high, medium-low, and low-income groups, respectively.

## Discussion

We aimed to quantify how provider-related factors are associated with patients’ revealed, GP-mediated choice of arthroplasty provider in Norway, expressed as WTT in minutes, and to explore how WTT for travel time was modified by patient characteristics. We found that provider attributes associated with the greatest WTT included dedicated elective capacity, higher surgical volume, shorter waiting times, receiving treatment within health region of residence and the presence of an adult child living near the provider. In contrast, longer travel time was associated with steeper individual-level disutility for older, sicker, less educated, and lower-income patients.

Only 6% of patients received care outside their home health region, indicating formal free choice is exercised by a minority and is socially and geographically patterned. The proportion varied from 0.6% to 31% across the health enterprise referral areas, being highest in the northern and most peripheral areas and lowest in the capital region. This suggests that the use of free choice is geographically patterned, with patient flows directed from peripheral areas towards larger, more centrally located providers.

Rather than a purely autonomous patient choice, we observed the outcome of a joint, GP-mediated decision. Norwegian patients reach a hospital only after a GP referral, and the GP’s knowledge of local capacity, waiting times, and provider quality co-determines the choice set the patient effectively faces. Our WTT estimates therefore quantify revealed preferences expressed through this referral filter and may reflect some GP behavior and selective referral as well as patient preference. The same remark applies to the socioeconomic and age-related gradients, which may partly arise from differential GP guidance to patients with lower health literacy or fewer resources to navigate the choice.

The WTT framework quantifies this heterogeneity in patient-centered terms where most patients exhibit moderate travel-time tolerance, but a long tail of patients accept considerably longer travel for clinically meaningful provider features.

The prominence of structural factors aligns with earlier, smaller studies [1,3], and our analysis quantifies their relative contribution on the patient-relevant WTT scale. The association between surgical volume and choice (mean WTT 16 min, CI 15–17) may reflect both patient perceptions of expertise and GPs’ awareness of volume–outcome relationships [21]. The very large mean WTT for a dedicated elective surgical unit (156 min) and for staying within the home region (70 min, CI 67–72) indicate that, on the population scale, these are the most decisive provider features, but the gap between the mean and the median WTT for the elective unit (8 min) shows that the population mean is driven by a subgroup with very high travel-time tolerance rather than by a uniform population effect. These observations are consistent with reports by Victoor et al. [1] and Aggarwal et al. [3]. The proximity to an adult child near the provider is a new and interesting finding, which confirms observations in our recent qualitative study [6], and may reflect the value of informal support and reduced logistical burden. A pattern of migration from rural Norway to the capital region may partly explain this geographic co-variation.

The socioeconomic gradient in WTT mirrors findings from England and Denmark [22,23] and underscores that universal entitlements do not automatically yield equal realized access. Lower-income and lower-education groups appeared more tightly bound to their local hospital, in keeping with higher indirect costs, fewer resources, and a more GP-directed referral path [24]. The change in the age-travel-time relationship around 67 years may relate to retirement, altering time costs and flexibility. In Norway, patients choosing a provider outside their health region may incur deductibles (circa €70 in 2015) and partial self-coverage of lodging during travel, which may constrain choice for some. Removing or reimbursing these costs for patients who must travel could reduce financial barriers and improve access.

Education gradients in WTT could reflect differences in health literacy or access to information. Health literacy likely shapes patients’ ability to navigate provider choices. A Norwegian study showed that one-third of arthroplasty patients had low digital health literacy [25]. Individuals with limited health literacy may struggle with complex choice sets and comparative information and may rely more on GP advice rather than actively searching for provider information [6,24]. Simplifying choice architectures (e.g., decision aids) and equipping GPs to tailor guidance may mitigate informational barriers, as shown in a qualitative study [6].

Norway’s already decentralized hospital structure limits the potential to reduce travel time by adding more providers [8]. Expanding dedicated elective capacity in the largest cities in each region, together with shorter waiting times, could shift trade-offs in favor of within-region care, particularly within the Northern Regional Health Authority where cross-region utilization is highest. Modifiable provider features like waiting-time transparency, dedicated elective capacity, and out-of-pocket travel costs are potential policy levers, but the observational design precludes any causal claim regarding the size or direction of their impact on choice.

### Limitations

First, the GP referral filter means associations may reflect some GP behavior and selective referral as well as patient preference. Second, provider attributes were averaged over the study period because annual data was not consistently available; this smooths out near-term variation in waiting times and volume and might bias coefficients towards the null. Third, median dichotomization of waiting time and surgical volume may have obscured non-linear patterns. However, sensitivity analysis using 3 and 4 categories for waiting time and surgical volume showed the main conclusions remain unchanged. Fourth, the 2-stage analysis does not propagate first-stage uncertainty and may underestimate second-stage standard errors. The multilevel results should therefore be interpreted as associations with estimated travel-time preferences, conditional on the first stage mixed logit model. Fifth, personal after-tax income does not capture household composition, so the income gradient reflects individual rather than disposable household income. Finally, this study is based on observational data; residual confounding due to unmeasured provider features (surgeon reputation, marketing) and patient features (health literacy) may remain. The results should therefore be interpreted as associations rather than causal effects.

### Conclusion

Norwegian patients’ revealed, GP-mediated choice of arthroplasty provider was associated with provider attributes that translate into clinically interpretable willingness to travel. The willingness to travel was modified by patient characteristics, with older, sicker, lower-income, and lower-educated patients tolerating less additional travel. Elective care, higher surgical volume, and adult children near the provider were also associated with higher willingness to travel. This indicates that realized choice might be shaped by both modifiable structural features of the healthcare system and by socioeconomic gradients.

### Supplementary data

Supplementary Tables S1 is available as supplementary data on the article page, doi: 10.2340/17453674.2026.46430

## Supplementary Material



## Notes

BKH: Devised the research program and protocol. Designed the study. Acquired, analysed, and interpreted the data. Drafted, edited, and revised the manuscript. CK: Devised the research program and protocol. Designed the study. Analysed and interpreted the data. Drafted, critically edited, and revised the manuscript. YT: Designed the study. Analysed and interpreted the data. Critically edited and revised the manuscript. AMF, ES, OF: Designed the study and interpreted the data. Critically edited and revised the manuscript. TI: Devised the research program and protocol. Designed the study and interpreted the data. Critically edited and revised the manuscript.

Handling co-editors: Li Felländer-Tsai and Robin Christensen

*Acta* thanks Michael Axenhus and Alexander Oxblom for help with peer review of this manuscript.
