# Supplementary material for: Patients’ choice of healthcare provider for primary hip and knee arthroplasty: a national register-based observational cohort study of 62,676 patients from Norway, 2014–2018
Source: Acta Orthop. 2026 Aug 31;97:561–7. doi: 10.2340/17453674.2026.46430 (PMC13527609; doi:10.2340/17453674.2026.46430)
Supplement: Supplementary file 1 [file ActaO-97-46430-s1.pdf]

Supplementary data

Supplementary Table S1. Characteristics of the 51 Norwegian hospitals providing publicly funded primary hip or knee arthroplasty during 2014–2018

Arthroplasty providers	Surgical volume	Waiting time	Elective unit
Finnmark Hospital Trust, Kirkenes Hospital	Low	High	No
Finnmark Hospital Trust, Hammerfest Hospital	Low	High	No
University Hospital of North Norway, Tromsø Hospital	High	High	No
University Hospital of North Norway, Harstad Hospital	Low	High	No
University Hospital of North Norway, Narvik Hospital	Low	High	No
Nordland Hospital Trust, Vesterålen Hospital	Low	Low	No
Nordland Hospital Trust, Lofoten Hospital	Low	High	No
Nordland Hospital Trust, Bodø Hospital	Low	High	No
Helgeland Hospital Trust, Mo i Rana Hospital	Low	High	No
Nord-Trøndelag Hospital Trust, Namsos Hospital	High	Low	No
Nord-Trøndelag Hospital Trust, Levanger Hospital	High	High	No
St. Olavs Hospital Trust, St. Olavs University Hospital	High	High	No
St. Olavs Hospital Trust, Orkdal Hospital	High	Low	No
St. Olavs Hospital Trust, Røros Hospital	Low	Low	No
Møre and Romsdal Hospital Trust, Molde Hospital	Low	Low	No
Møre and Romsdal Hospital Trust, Kristiansund Hospital	High	High	No
Møre and Romsdal Hospital Trust, Ålesund Hospital	High	High	No
Møre and Romsdal Hospital Trust, Volda Hospital	High	Low	No
Førde Hospital Trust, Førde Hospital	High	High	No
Førde Hospital Trust, Lærdal Hospital	Low	High	Yes
Bergen Hospital Trust, Haukeland University Hospital	High	High	No
Bergen Hospital Trust, Kysthospitalet i Hagevik	High	Low	Yes
Bergen Hospital Trust, Voss Hospital	Low	Low	No
Haraldsplass Hospital	High	Low	No
Fonna Hospital Trust, Stord Hospital	Low	Low	No
Fonna Hospital Trust, Haugesund Hospital	High	High	No
Stavanger Hospital Trust, Stavanger University Hospital	High	High	No
Haugesund hospital for rheumatic diseases	Low	High	Yes
Vestre Viken Hospital Trust, Drammen Hospital	High	Low	No
Vestre Viken Hospital Trust, Bærum Hospital	High	Low	No
Vestre Viken Hospital Trust, Ringerike Hospital	High	Low	No
Vestre Viken Hospital Trust, Kongsberg Hospital	High	Low	No
Telemark Hospital Trust, Skien Hospital	High	Low	No
Telemark Hospital Trust, Notodden Hospital	Low	Low	No
Akershus University Hospital	High	Low	No
Akershus University Hospital, Kongsvinger Hospital	High	Low	No
Innlandet Hospital Trust, Elverum Hospital	High	Low	No

Innlandet Hospital Trust, Gjøvik Hospital	High	Low	No
Innlandet Hospital Trust, Lillehammer Hospital	Low	Low	No
Innlandet Hospital Trust, Tynset Hospital	High	Low	Yes
Oslo University Hospital, Ullevål Hospital	High	High	No
Oslo University Hospital, Rikshospitalet	High	High	No
Østfold Hospital Trust, Østfold Hospital	High	High	Yes
Sørlandet Hospital Trust, Kristiansand Hospital	High	High	No
Sørlandet Hospital Trust, Arendal Hospital	High	High	No
Sørlandet Hospital Trust, Flekkefjord Hospital	Low	High	No
Vestfold Hospital Trust, Larvik Hospital	High	Low	Yes
Diakonhjemmet Hospital	High	Low	Yes
Lovisenberg Diaconal Hospital	High	High	Yes
Martina Hansens Hospital	High	Low	Yes
Betanien Hospital	Low	Low	Yes

Hospitals are classified by annual surgical volume of hip or knee arthroplasties (High ≥ 109/Low < 109 procedures per year, dichotomized at the median), by reported waiting time to surgery (High ≥ 10.2/Low < 10.2 weeks, dichotomized at the median), and by whether the provider operates a dedicated elective surgical unit (Yes/No), defined as orthopedic surgical capacity reserved for elective procedures and protected from emergency interruptions.
